# Our Periodical

**Published:** 1835

**Authors:** 


### III.—ORIGINAL.

---

### The Western Journal.

---

*E Sylvis Nuncius.*

---

CINCINNATI, JANUARY 1836.

---

### Art. I.—Our Periodical.

At the moment when our last number was about to issue from the press, we found ourselves turned over to new publishers, which to an editor is a kind of turning over of a new leaf. We should have published the fact to our distant friends, if a single page of our last sheet had remained open. Since we commenced editing this work (the oldest periodical journal in the West,) in 1827, we have been so often turned over, that it seems to us as matter of astonishment, that we have not been overturned. Counting on our fingers (in any thing but a poetical reverie) we find, that already, before the completion of our ninth volume, we have owed allegiance to nine publishers! Thus, if our editorial vitality had not been truly feline, we should now be defunct. We are far from complaining of these vicissitudes, seeing, that as meteorologists, we were long since instructed in the necessity of change to preserve the purity of the atmosphere. Nor are we inclined to quarrel with the gentlemen who have transferred us, howbeit, we are well known to be an humble but ardent member, of the *genus irritabile.* If we have run the gauntlet, no malign torture has been inflicted on us; and, we have at last the consolation to know, that those for whom we cater, are highly respectable in *numbers*, and not less so, as they themselves will admit, in professional attainments and inquisitiveness. But we *do* complain of the *effects* of these rapid mutations, on the regularity of our publication. It has never been issued with the promptness which we know to be acceptable to readers, and ought to be regarded as a duty both by editors and publishers. Some cynic has said, that he who is good at apologies is seldom good at any thing else; and we are not without apprehension that the apothegm may be applied to us. Should such an application be made, we shall certainly plead guilty, for common candor would require it. To avoid this, we shall not venture on another apology nor make another promise, lest we should be compelled to look back with chagrin to its violation. As to the *present* past, why it is past, and wherefore recall it? We are not of the retrospective temperament, but prefer to live with the future: to look forward, instead of backward: to enter into imaginative sympathy with those who are to come, rather than with those who are gone: to watch the growth and development of the generation that is sprouting up around us—consider the wants of their professional minority—investigate the causes which may retard their intellectual expansion—and, now and then, write a hygienic prescription, designed to promote their mental health. Having this decided taste for communion with the young, and, at least *professing*, to feel a deep interest for their prosperity, it is a pleasant circumstance, that a majority of our readers belong to that stage of human life. We rely much on the magnanimity of that era, and assume that it will pardon many remissnesses, which a more advanced and querulous period, would condemn: we confide in its modesty, and shall, therefore, believe it ready to receive admonition. Now, having placed ourselves for a while on our editorial *confessional*, we shall fancy a goodly number of our readers, to mount the chair, and give some account of *themselves.* In reply to the question, why they do not observe, experiment, register, and write for the journal which they patronize as readers, what can they say? One answers, that he has not time—no, indeed, his business is too extensive;—he sees and does so much, of that which ought to be recorded and published, that he has no leisure for either! Another is unworthy to appear in print;—*his* imperfect observations could add nothing valuable, to the stock of facts already accumulated—*his* powers of intellectual analysis can decompose nothing intricate—his excursions can lead the profession into no unexplored region! To such an one we say, it requires *more* talent, to practice the profession successfully, than to report the results to the world. Another cannot write, because, his professional business not being quite large enough, he has superadded something else, that he might "make money," a little faster:—this has diverted his thoughts from science—the hours not employed in visiting the sick of his town or village, must be devoted to an inspection of vacant lots and dilapidated houses! Speculations in land have supplanted speculations in philosophy, and avarice has asphyxiated ambition! But such an one has already lost *caste* in his profession—he has sunk to a lower level in society—and should have the sense of propriety and duty, which would lead him to withdraw from that which his personal presence cannot but desecrate! Others, again, intend to write when they get older. They may, we admit, *publish* when they get older, but they must write when young; and we have observed, that in general, those who do not publish do not write. A young physician may distrust his powers of philosophical analysis, his ability to trace out new principles, his capacity for arranging them into new systems, but no young physician, need to doubt his ability to observe and report new cases and facts, and these are as likely to present themselves to the young as old. Away then, we say, with these various excuses, and let all who would see the medical profession of the West, ascend with the general structure of society, come forward and raise that profession, and themselves, and the WESTERN JOURNAL, with the contribution of every thing that is valuable in their experience. Thus they will instruct each other, and make our periodical, when it wanders, out of the Great Valley, what it professes to be—E SYLVIS NUNCIUS.

